# Assisted death for prisoners and forensic patients: complexity and controversy illustrated by four recent cases

**DOI:** 10.1192/bjb.2024.23

**Published:** 2024-10

**Authors:** Roland M. Jones, Alexander I. F. Simpson

**Affiliations:** 1 Centre for Addiction and Mental Health, Toronto, Canada; 2 University of Toronto, Toronto, Canada

**Keywords:** Euthanasia, assisted suicide, psychiatry and law, prison, correctional centre

## Abstract

Medical assistance in dying (MAiD) (which includes euthanasia and assisted suicide) is available in an increasing number of countries. In Belgium, The Netherlands and Switzerland (and was due to be implemented in Canada from 2024) eligibility includes mental suffering in the absence of any physical disorder. There are particular ethical and legal issues when considering MAiD for those involuntarily detained in prisons and hospitals. We describe four recent cases that illustrate these complexities, and highlight issues of equivalence of healthcare and self-determination against concerns about the criteria for determining eligibility of those with non-terminal conditions as well as the objections raised by victims and families and the demands for justice.

Medically assisted death is legally sanctioned in an increasing number of countries and jurisdictions.^1^ It might involve a drug administered at a lethal dose by a physician (euthanasia) or given to the person to administer themselves (assisted suicide). For the purpose of this paper we will use the term medical assistance in dying (MAiD) to include both euthanasia and assisted suicide.

In most countries that allow MAiD, the individual is required to be in the final stages of an illness or disease in which there is unbearable suffering with no hope of recovery and the death is foreseeable within months. However, some countries, notably Canada, Belgium, The Netherlands and Switzerland, allow MAiD for individuals who have conditions that are not terminal, but are nevertheless experienced by the individual as unbearable, for which there is no other remedy to their suffering.

For those countries that permit MAiD for non-terminal illness, a further distinction can be drawn between those permitting MAiD for medical conditions only and those that permit MAiD for mental suffering as the sole criterion (or the cause of suffering is not specified). Belgium permits MAiD for those that have a ‘medically futile condition of constant and unbearable physical or mental suffering that cannot be alleviated, resulting from a serious and incurable disorder caused by illness or accident’.^2^ In Belgium therefore, the condition, either mental or physical, must arise from accident or illness and be incurable but not necessarily terminal. The Netherlands permits MAiD for those whose ‘suffering is unbearable with no prospect of improvement’;^3^ thus a particular origin of the suffering is not specified and does not need to be categorised as a medical condition. Switzerland permits assisted suicide to any competent adult, provided they can at least trigger the infusion of drugs themselves, irrespective of reason for doing so.^4^ In Canada the 2016 legislation permitted MAiD only for terminal conditions^5^ but was amended in 2021 to include non-terminal conditions.^6^ However, MAiD in which the sole medical condition is a mental disorder was delayed and was due to be introduced in March 2024. Then, on 1 February 2024, the Canadian government announced that MAiD for those in who mental disorder is the sole medical condition would be delayed for a further 3 years. Currently in Canada, two independent physicians or nurse practitioners are required to determine eligibility, first that the person has the capacity to make the decision and second that the person has a ‘grievous and irredeemable’ condition. Psychiatrists do not have a designated role, but can participate as an independent physician. If the legislation proceeds to include MAiD for chronic mental disorders, psychiatrists would likely be called on as having expertise in the condition that is causing the person's suffering to provide either one of the MAiD assessments or consultation to those undertaking the assessment.

There has been debate about the extent to which prisoners should have access to MAiD among those countries where MAiD is permitted. The arguments in favour of allowing access have often been centred on self-determination and equivalence of care.^7–9^ Rule 24 of the United Nations Standard Minimum Rules for the Treatment of Prisoners (the Nelson Mandela Rules)^10^ articulates that prisoners should enjoy the same standard of healthcare as available in the community. This principal is not legally binding and most jurisdictions fall short of this. For example, the 1992 Corrections and Conditional Release Act in Canada stipulates that prisoners should be provided with ‘essential health care’ that ‘conform[s] to professionally accepted standards’, and the European Court of Human Rights finds that the health of prisoners must be ‘adequately ensured by providing them with the requisite medical assistance’.^11^ Nevertheless, the intention is to ensure that citizens have access to treatments and health services irrespective of incarceration status. In some jurisdictions, MAiD is considered to be healthcare, so the principle of equivalent access to healthcare applies to people who are incarcerated (that MAiD is healthcare is arguable, but will not be considered further here). In this paper we describe four recent cases that raise questions about the complexity of voluntariness when the person is incarcerated and the issues of wider demands of justice.^8^ All material in these case summaries is from public records.^12^

## Case 1: Marin Sabau (Spain)

On 14 December 2021, Marin Sabau, a 45-year-old single security guard went into his workplace of over 10 years, wearing a disguise and armed with a pistol and silencer, and shot and injured three staff. Over an 8-year period, he had lodged multiple complaints against his employer, alleging breaches of his employment rights. After the shooting, he drove to a nearby shopping centre, where he sat in his car and sent a 3500-word email to company senior staff as well as the victims, stating ‘I don't want to kill them … I'm not crazy. I have it all perfectly planned’.^12^ He added ‘Lessons learned with blood are not quickly forgotten’.^12^

Less than 2 h after the shooting, police saw him driving, and three officers stopped him at a roundabout. M.S. shot an officer in the arm after he was challenged to throw away his gun. Shots were exchanged and M.S. drove away. Police followed him to a farm, surrounded him and shot him at least three times. He was airlifted to hospital unconscious. He had fractures of the skull, neck and ribs and a spinal cord injury and badly injured arm. He regained consciousness after 3 weeks, and had multiple surgeries, including a leg amputation.

On 20 June 2022, it became public that M.S. had requested MAiD. Doctors confirmed that he met Spanish legal criteria for MAiD. On 11 July 2022, he told criminal investigators that he was paraplegic, had 45 stitches in his hand, could not move his left arm and could not feel his chest.

Victims in this case expressed their objection that he would be allowed to die without being tried for his offences. Public prosecutors also opposed M.S.'s application for euthanasia, arguing that the victims had a right to a trial. A lawyer on behalf of one of the victims said ‘He has the right to a dignified death, of course, but what about the compensation of the victims?’^13^

M.S. was euthanised on 28 July 2022. Less than 8 months had elapsed since the shooting, and M.S. had not been convicted of any offences. One of the victim's lawyers stated the decision to allow MAiD ‘hasn't taken into account the victims’ suffering nor their dignity’.^13^

## Case 2: Frank Van Den Bleeken (Belgium)

Frank Van Den Bleeken was one of six children. He was placed in a care home when he was aged 6. He was reportedly abused as a child and raped when he was aged 15. He was first imprisoned for committing sexual offences at the age of 21. Following his release, he raped and killed a 19-year-old woman. He was found not criminally responsible for this offence and received treatment in a psychiatric wing of a prison. Seven years later he was released and he attacked three more victims within weeks of his release; they were aged 11, 17 and 29. He was then sentenced to life in prison.

In 2011, after 30 years in custody, he requested euthanasia, stating that he had been denied psychiatric help and was experiencing unbearable suffering. He recognised that he still had images of violent sexual behaviour, that he remained a danger to others and that life in jail was causing him ‘unbearable psychological suffering’.^14^ He said ‘I am a danger to society … What am I supposed to do? What is the point in sitting here until the end of time and rotting away? I would rather be euthanized’.^14^ He stated ‘If people commit a sexual crime, help them deal with it. Just locking them up helps no one: not the person, not society and not the victims. I am a human being, and regardless of what I have done, I remain a human being. So yes, give me euthanasia’.^14^ His request for euthanasia was rejected, stating that every possible treatment must be considered first.

A family member of one of the victims also objected, stating ‘For us this is incomprehensible. He should rot in his cell … We hear his lawyer say on the radio how much their client suffers. Well, we are suffering too’.^15^ She said ‘Commissions, doctors and other experts have investigated our sister's killer. But during all these years, not a single commission has examined our case. Not a single doctor or expert has asked us how we are doing now’.^15^

In September 2014, after 3 years of appeal, by then aged 52, the court granted F.V.D.B. MAiD and he was to be euthanised in jail on 11 January 2015. However, although the court granted it, the doctor who was going to carry the procedure announced their decision ‘to no longer continue the euthanasia procedure’,^16^ and he was instead transferred to a prison psychiatric facility.

## Case 3: Geneviève Lhermitte (Belgium)

Geneviève Lhermitte was born in Belgium in November 1966. She married and had five children and worked as a French and history teacher. After the birth of her first child, she suffered with depression and was unable to work. In June 2004, at the age of 33, she started receiving treatment from a psychiatrist and was prescribed antidepressants, anxiolytics and hypnotics. From September 2006 she was seeing a psychiatrist every 3 weeks and was reporting increasingly low mood. On 27 February 2007, she wrote again to her psychiatrist, saying ‘I have not felt well these last few days. I'm having dark thoughts. They are suicidal thoughts which are going to carry me away and I will take my children with me. It's a daily struggle. There is no solution to my problem […] I imagine scenarios which are both true and realistic and I know I am capable … ’.^17^ On 28 February, she put a bag of jewellery and a letter through the letterbox of her friend stating ‘I have decided to go a long way away with the children forever’.^17^

She killed her five children, aged 15, 12, 10, 7 and 3, one by one, by slitting their throats. She then attempted suicide but survived. During the investigation, a panel of three court-appointed psychiatrists concurred that she suffered from major depression and recommended that she be found not criminally responsible. The jury did not accept the expert testimony and she was sentenced to life imprisonment. She served 12 years in prison and in 2019 she was given a conditional release on the condition of psychiatric treatment for severe depression. After this treatment she could be released. While in the hospital, she tried to take her own life. In 2022 she asked for euthanasia on the grounds of unbearable psychological suffering. A request was granted and on 28 February 2023 she was euthanised. Her mother said her daughter had been in unbearable pain for 16 years. She said ‘Certainly, Geneviève was helped by psychiatrists during these years; she was listened to by the medical profession, and she was taking medication. But all this never gave her back her children. That was what her suffering was, having lost her children. Her goal was to join them’.^18^

## Case 4: Peter Vogt (Switzerland)

Peter Vogt was sentenced to 10 years in prison in 1996, then aged 42, for multiple charges of rape, including against a child. In 2004 legal provision under Swiss law allowed prisoners with ‘sexual delinquencies’ to be held indefinitely and he was effectively given an indeterminate sentence. In July 2018, he applied for assisted suicide, citing both physical and mental reasons. He had kidney and heart disease. He said ‘Nobody should have to commit suicide in his cell alone [ … ] It is natural that one would rather commit suicide than be buried alive for years to come’ and ‘It would be better to be dead than to be left to vegetate behind these walls’.^19^ Following review, it was determined that prisoners had the right to an assisted suicide under certain conditions, and he received euthanasia on 28 February 2023.

## Discussion

The framework that supports MAiD for prisoners with terminal conditions is understandable on the basis of equivalence of access to healthcare. However, there are other ethical concerns when considering situations in which those incarcerated have no physical disorder. In three of the cases outlined above, the main reason for requesting MAiD was suffering due to mental causes, and in the other, it appeared that physical injury was the main cause of suffering. F.V.D.B., G.L. and P.V. all cited the cause of their distress as being mental suffering; in one it was mental suffering as a consequence of their crimes (loss and guilt from killing her children), in the other two it was hopelessness caused by the punishment (incarceration without prospect of release). M.S. had severe physical injury, which appeared to be the reason cited as the cause of his suffering.

Proponents of MAiD for mental disorders as the sole underlying condition argue that excluding mental disorders is discriminatory and stigmatising of those with mental disorders. Some mental conditions, such as schizophrenia, bipolar disorder and schizoaffective disorder, more easily fit by analogy to physical disorders (having a recognised aetiology even if not fully understood, with recognised symptoms and signs, treatments and prognoses). The argument is not one of whether individuals with mental disorders experience suffering at least as much as those with physical conditions, nor that they should not be eligible for treatment or services, but whether the cause of the suffering should be considered irrelevant in a framework that grants autonomous requests to end a person's life.

### Suicide and self-determination

Suicide is not a criminal act in most countries, and although principles of autonomy and self-determination are generally considered to be fundamental rights, organisations (particularly those that restrict autonomy, such as hospitals and prisons) are required to go to great lengths to assess risk of suicide and to prevent suicide of individuals under their care. There are indeed legitimate concerns about ensuring that prisoners’ rights are upheld and that they have equivalence in access to healthcare and procedural fairness. Unrestricted self-determination and equivalence means giving incarcerated individuals the means and assistance to suicide. Lawmakers, and society more generally, will need to consider carefully whether this is the end-point that is desired in the pursuit of equality and self-determination. For instance, the Swiss authorities recently ruled in this case that there was no legal authority to deprive P.V. of the right to assisted suicide that would be available to him in the community (though of note, it was his confinement that was causing his wish to die). Suicide is already the leading cause of death among prisoners.^20,21^ A review of studies found that 9–13% of prisoners reported having made a suicide attempt at some point during their incarceration, and 24% of prisoners had considered suicide in the previous 12 months.^22^ Providing means and access to suicide among prisoners who request it would undoubtedly have high take-up if it were to be provided with limited restrictions. Lawmakers and society would need to be aware of and comfortable with this prospect.

### How do we define suffering, and when is it irremediable?

In two of the above cases (F.V.D.B. and P.V.), the individuals appraised the circumstances of their lives and determined that death was preferable to imprisonment. F.V.D.B. stated ‘ What’s the point in sitting here until the end of time and rotting away? I’d rather be euthanised’; and P.V. said that he would rather be dead than ‘be left to vegetate behind these walls’.^19^ G.L. did not wish to continue living after killing her children, irrespective of incarceration status, and had attempted suicide before being granted MAiD. Although depression can lead to nihilistic thoughts, the decision to end one's life may also be a rational one. But if access to MAiD is to be restricted (and we believe it should) and the appraisal as to suitability to access it falls to others to decide on the basis of irremediable suffering, how is suffering defined and when is it irremediable?

Pain and suffering are overlapping but distinct concepts, and it is worth considering the difference between them as they might apply to MAiD. Pain is defined as an unpleasant experience, which may have physical or psychological origin and incorporates the person's interpretation, cognitive awareness, personality or disposition, as well as cultural and educational factors. Suffering is an ‘unpleasant or even anguishing experience which can severely affect a person on a psychological and even existential level’.^23^ Pain is not necessarily experienced as suffering, and suffering is not necessarily caused by physical pain. Although pain appraised from a medical model may be amenable to ‘treatment’, alleviation of suffering fits less well within the medical model, which focuses on diseases, treatments and cures, rather than existential issues of meaning and quality of life.

Suffering can be considered to be a dynamic model, made up of a variety of possible causes and mitigating factors. The perception of pain, and whether it leads to suffering, is an individual experience and can vary depending on the individual's own resilience and coping, the presence and degree of social and personal support, as well as pain management treatments. MAiD is predicated on the presence of irremediable suffering. While the underlying cause of the pain may not be modifiable (in the case of M.S., who had loss of limb and paralysis), the suffering arising from it may well be modifiable. It is difficult therefore to appraise whether suffering is, and will always remain, irremediable.

F.V.D.B., G.L. and P.V. all suffered from psychological pain, although in each case there may be differences in the extent to which the suffering could be alleviated. G.L. appeared to have had extensive treatment, including hospital admission. As her mother stated, ‘Geneviève was helped by psychiatrists during these years; she was listened to by the medical profession, and she was taking medication. But all this never gave her back her children’. F.V.D.B. complained that he did not have access to sufficient treatment. He stated ‘If people commit a sexual crime, help them deal with it. Just locking them up helps no one’. After being granted permission for MAiD and the procedure was not carried out, he was transferred to a hospital, whereupon there have been no further requests for MAiD, and therefore his suffering appears not to have been irremediable and options to alleviate it were manifestly not exhausted.

Although accepting the limitations of a biomedical model of suffering, an individual's suffering can change over time owing to adaptation and by improving support, coping and resilience. How an individual copes with life-changing circumstances therefore depends on a number of personality and environmental characteristics, as well as the severity of functional impairment.^24^ Prisoners in general have heightened psychological distress in the early part of incarceration, and the severity of psychological distress, severity of symptoms of mental disorder and the proportion of inmates reaching diagnostic threshold for mental disorder all reduce over time during incarceration for most inmates.^25–29^ Decisions about tolerability and irremediability of suffering may therefore be judged differently by the individual at the early part of incarceration compared with later on in the incarceration. Similar to the initial shock of incarceration, and relevant to the case of M.S., sudden disability can be associated with significant psychological distress, following which there may be a pattern of acceptance and even positive psychological change.^30,31^

It is difficult therefore for anyone to conclude that a person's suffering is, and will always be, irremediable. In addition, the cause of suffering, as in two of the cases (M.S. and P.V.), can be dissatisfaction with quality of life in prison. Given that the state's purpose in incarceration is to deliberately impair quality of life by restriction of liberty and confinement, it is perhaps at least ironic that the suffering caused by incarceration, especially indefinite incarceration, can then be the qualification for an assisted death. Authorities will need to consider whether this is the balance they want to strike between the right to punish and the right to self-determination.

### Does MAiD undermine the principles of justice?

In two of the cases (M.S. and F.V.D.B.) there was vocal opposition to MAiD from victims or their families on the basis that the person's death would deprive them of a right to justice and thus ignored their own suffering.

There has been a growth in victims’ participatory rights in criminal proceedings in many countries, particularly in the past two decades. The Council of the European Union adopted legislation in 2001 setting out minimum standards for victims of crime, including the right to be heard and to participate,^32^ and a Department of Justice Canada report in 2021 stated ‘It is clear that providing crime victims with some degree of control and autonomy is an important first step in the healing process. Victim participation is the first step in regaining self-esteem lost as a result of criminal victimization’ (p. 16).^33^ The reality, however, may be that there is more limited impact in decision-making. The report states:

‘in 2012, the B.C. [British Columbia] Provincial Court noted that “The sentencing process is fundamentally incapable of dealing with the legitimate needs of victims and their survivors” (*R v Smith* 2012:8), and in 2013 the B.C. Court of Appeal pointed out that “it must be remembered that a criminal trial, including the sentencing phase, is not a tripartite proceeding. A convicted offender has committed a crime – an act against society as a whole. It is the public interest, not a private interest, which is to be served in sentencing” (*R v Berner* 2013:16)’ (p. 9).^33^

In addition, an analysis of the use of victim impact statements in sentencing showed that the presence of a victim impact statement did not have any effect on the sentencing after adjusting for severity of offence, and that victims often felt re-traumatised by the experience of providing one.^34^

In the case of M.S., despite opposition from victims, the Spanish court ruled that there is no exception in law as to MAiD eligibility for those even if in the middle of legal proceedings. In her ruling on the matter, Judge Torres said ‘One could say that there is a clash of fundamental rights here. The right to dignity and personal autonomy is a fundamental right that trumps the victim's rights to a fair trial’.^13^

## Conclusions

Given the complexities of MAiD in correctional settings, jurisdictions seeking to adopt it in some form, or those reconsidering it as it applies to prisoners, need to consider several issues. The following points sum up our opinion on the matter.

- Prisoners with a terminal illness should be permitted equal access to MAiD (when otherwise permitted in the community) using the principles of equivalence of healthcare.
- If policymakers and lawmakers wish to extend assisted suicide for prisoners as a right of self-determination, they should not be absolved of their obligations to provide humane environments for detention and must provide the fullest options for medical and psychological therapies. They would also need to acknowledge and be prepared for large numbers of prisoners seeking this route of death.
- MAiD should not be extended to those with non-terminal conditions under the guise of healthcare. MAiD for non-terminal conditions and suicidality are indistinguishable. If the overriding principle is right to autonomy, then having the autonomous right to suicide does not entail the right to have one's life terminated by a physician on request.
- Although many countries increasingly provide victims of crime with avenues to participate in the legal proceedings, it needs to be acknowledged that such participation has limited impact on outcomes. In approving MAiD, the rights of victims to participate will be overridden by the prisoner's right to MAiD.
- Jurisdictions that are considering (and do not already have) laws permitting MAiD for non-terminal disorders should consider where the balance between personal autonomy and paternalism should lie. Those in favour of personal autonomy being the primary and overriding value will conclude that citizens have the right to choose when to die. Those in favour of more paternalism would argue that laws are needed to protect and support the vulnerable in society and to prevent the killing of others under all circumstances. Should a policy that pursues personal autonomy as the primary value be preferred, we recommend that the decision to end one's life should be an individual decision, not a healthcare decision, and should therefore not be made by a doctor or other healthcare practitioner. Instead, as in a current proposal in the UK,^35^ particularly for those who are incarcerated, we recommend that the decision should be placed with a judge who can authorise assisted dying. Alternatively, the approach in Switzerland is most consistent with the principles of autonomy and does less to distort the medical role as it allows for any competent adult to request suicide which is administered by non-medical providers. Thus autonomous decisions can be made without value judgements by healthcare practitioners as to whether severity and irremediability of suffering qualifies the person to be allowed to die. Nevertheless, while this may be acceptable in the community, the extension to use in the prison setting is inconsistent with the duty of care that the state has to protect and maintain the well-being of those it detains, and in our view is ethically unacceptable.

## Data Availability

Data availability is not applicable to this article as no new data were created or analysed in this study.

## About the authors

**Roland M. Jones**, MBChB, BSc, MSc, FRCPsych, PhD, is a forensic psychiatrist and scientist at the Centre for Addiction and Mental Health, Toronto, Canada, and an Associate Professor in the Division of Forensic Psychiatry at the Department of Psychiatry, University of Toronto, Canada. **Alexander I. F. Simpson**, MBChB, BMedSci, FRANZCP, FCPA, is a senior scientist at the Centre for Addiction and Mental Health (CAMH), Toronto, Canada, holding the Research Chair in Forensic Psychiatry at CAMH and the University of Toronto, Canada, and Professor in the Department of Psychiatry, University of Toronto, Canada.
