# Assisted death for prisoners and forensic patients: complexity and controversy illustrated by four recent cases – CORRIGENDUM

**DOI:** 10.1192/bjb.2026.10226

**Published:** 2026-06

**Authors:** Roland M. Jones, Alexander I. F. Simpson

**Keywords:** Euthanasia, assisted suicide, psychiatry and law, prison, correctional centre, corrigendum

In the original publication of this article, it was claimed that Peter Vogt received euthanasia on 28 February 2023 (p. 302). The authors have since realised that this is incorrect, and this has now been removed from the article.

The authors apologise for this error.
